# Characteristics of suicidal emergency room patients before and during the COVID‐19 pandemic in Japan

**DOI:** 10.1002/npr2.12341

**Published:** 2023-04-25

**Authors:** Kyohei Otani, Ryohei Yoshikawa, Atsumi Naitou, Haruko Fukushima, Kunitaka Matsuishi

**Affiliations:** ^1^ Department of Psychiatry Kobe City Medical Center General Hospital Kobe Hyogo Japan

**Keywords:** adolescent, hospitals, inpatients, mental health, suicide

## Abstract

**Aim:**

Owing to the stress caused by the behavioral restrictions and lifestyle changes during the COVID‐19 pandemic, suicide rates have increased in Japan, especially among young people. This study aimed to identify the differences in the characteristics of patients hospitalized for suicide attempts in the emergency room, requiring inpatient care before and during the pandemic over 2 years.

**Methods:**

This study was a retrospective analysis. Data were collected from the electronic medical records. A descriptive survey was conducted to examine changes in the pattern of suicide attempts during the COVID‐19 outbreak. Two‐sample independent *t*‐tests, Chi‐square tests, and Fisher's exact test were used for data analysis.

**Results:**

Two hundred one patients were included. No significant differences were found in the number of patients hospitalized for suicide attempts, average age, or sex ratio before and during the pandemic periods. Acute drug intoxication and overmedication in patients increased significantly during the pandemic. The self‐inflicted means of injury with high fatality rates were similar during the two periods. The rate of physical complications significantly increased during the pandemic, while the proportion of unemployed individuals significantly decreased.

**Conclusions:**

Despite studies predicting an increase in suicides based on past statistics of young people and of women, no significant changes were observed in this survey of the Hanshin‐Awaji region, including Kobe. This may have been owing to the effect of suicide prevention measures and mental health measures implemented by the Japanese government after an increase in suicides and after past natural disasters.

## Abstract 2

Despite studies predicting an increase in suicides based on past statistics of young people and of women, no significant changes were observed. This may have been owing to the effect of suicide prevention measures and mental health measures implemented by the Japanese government after an increase in suicides and after past natural disasters as well as infectious disease pandemics such as the H1N1 influenza.

## Graphical abstract

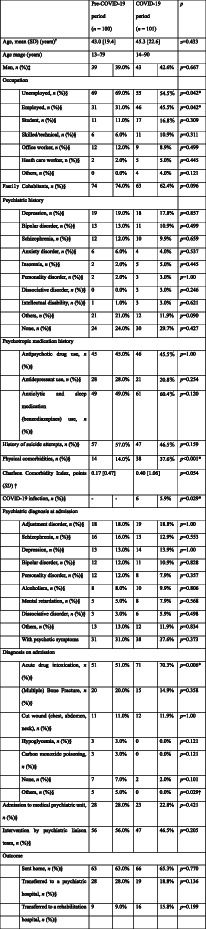

## 1 INTRODUCTION

COVID‐19 has led to a reported global increase in mental health episodes, particularly depression, anxiety, insomnia, and suicidal behavior.^1^, ^2^, ^3^, ^4^ Similar predictions have also been made in Japan.^5^ This is not a new phenomenon; previous pandemics also saw increased suicide rates, for example in the United States during the 1918–1919 influenza pandemic and among older adults in Hong Kong during the 2003 severe acute respiratory syndrome epidemic.^6^

Pandemics can affect economic downturns, increase unemployment rates, and lead to an increase in the rate of individuals with no previous history of mental illness attempting suicide.^7^

During the period from April 2020 to September 2021, the number of suicides per 100 000 population in Japan was 17% higher for men and 31% higher for women than predicted, based on trends from 2009 to 2019. Concerning the number of suicides by age group, the increase is more pronounced among young women and is reported to be 72% higher than the forecasted figure among women in their 20s.^8^ Other reports indicate that the number of suicides decreased in the first 4 months of 2020 but increased in the 17th month.^9^

While some studies have shown increased suicidal ideation in young people, other reports indicated lower self‐harm and suicide rates. Among young adults in Ontario, Canada, the first 15 months of the COVID‐19 pandemic were associated with a relative reduction in hospital treatment for self‐harm or overdosing.^10^ The number of people visiting emergency rooms in the United States for suicide attempts was significantly lower than before the pandemic, as were visits for overdoses and domestic violence.^11^ Additionally, a study among veterans, who are considered a high‐risk group for suicide attempts, reported a decrease in suicide rates after the pandemic.^12^ However, these lower rates may partly be due to hospitals being overwhelmed and reducing services for self‐harm and suicide.

This study aimed to examine changes in the general characteristics and patterns of people who attempted suicide and presented to the emergency room during the COVID‐19 pandemic (April 1, 2020–March 31, 2022), compared with those before the pandemic (April 1, 2018–March 31, 2020) in Japan.

The percentage of patients in the emergency department who attempted suicide in Japan was reported as 4.7% in a meta‐analysis to March 2013.^13^ It is important to formulate preventive measures for the emergency room where most people attempting suicide first arrive.^14^, ^15^ Therefore, we conducted an observational study examining the characteristics of patients who attempted suicide and were admitted to the emergency room before and during the COVID‐19 pandemic. Patients were examined by psychiatrists, assessed for risk, and provided with mental health support for post‐discharge care in collaboration with the emergency department and other physical medicine departments. Findings from this study may help formulate policies aimed at reducing suicide rates in emergency rooms.

## 2 METHODS

### 2.1 Participants

This study was a retrospective analysis. Data were collected from the medical records of patients admitted to Kobe City Medical Center General Hospital, the main hospital in Hanshin‐Awaji area, including Kobe, as emergencies and referred to psychiatrists.

We included patients who were admitted to the emergency room and provided with interventions by psychiatrists after they attempted suicide between April 1, 2018 and March 31, 2022. We excluded the following: patients who visited the emergency room for suicide attempts but were sent home because they promised not to reattempt suicide and did not require hospitalization, those with minor or no physical symptoms, those with severe psychiatric symptoms who were referred to psychiatric hospitals, and those who were confirmed dead after emergency transport. The interviews were conducted with verbal consent from a psychiatrist, an initial resident or late‐stage psychiatrist, and at least three medical staff members. At least one nonpsychiatrist in liaison team was included in the consultation to avoid bias in information and diagnosis. The interviews were conducted at the patient's bedside or in an interview room in the emergency ward, and there was no specific time limit. Questions were asked, including confirmation of the presence or absence of suicidal thoughts. To maintain the data confidentiality, data accumulation and statistical analysis were performed by a different person than the psychiatrist who examined the patient. An opt‐out method was used for inclusion in the study for patients to withdraw at any time; all patients agreed to take part in the study. Patients were not compensated for their participation. All procedures complied with the ethical standards of the relevant national and institutional committees on human experimentation and with the Helsinki Declaration. All procedures involving human patients were approved by the Research Ethics Review Board of the Kobe City Medical Center General Hospital.

### 2.2 Data collection and analysis

All research procedures complied with institutional practice guidelines, regulations, clinical research standards, and the principles of the Declaration of Helsinki. All data were encrypted, and the study protocol was approved by the Research Ethics Review Board of Kobe City Medical Center General Hospital (number: zn220702).

To compare the severity of comorbidities, we collected the following data from the electronic medical records: sex, age, COVID‐19 infection status, admission to a ward with physical complications, psychiatric liaison team intervention, the presence or absence of previous psychiatry visit and diagnosis, prescription history of antipsychotic, antidepressant, anxiolytic, or sleeping medication, the presence of physical complications, and Charlson Comorbidity Index (CCI) score.^16^ The CCI score was calculated using records from the Japanese Diagnosis Procedure Combination data.^17^

A descriptive survey was conducted to examine changes in the pattern of suicide attempts during the COVID‐19 outbreak. In addition, a cross‐sectional survey was conducted between the periods during and before the COVID‐19 pandemic to examine the number and methods of suicide attempts. All outcome variables were collected, stratified, and compared between the two periods: the “pre‐COVID‐19 period” and the “COVID‐19 period.” Two‐sample independent *t*‐tests, Chi‐square tests, and Fisher's exact test were used for data analysis. Significance was set at 0.05.

## 3 RESULTS

### 3.1 Patients' characteristics

During the fiscal year (FY) 2021, the number of patients admitted to the emergency room in the hospital was 9114. During the pre‐pandemic period, this hospital had approximately 30 000 emergency admissions per year. Mild to severe COVID‐19 patients have been accepted since March 3, 2020, when the COVID‐19 infection was first detected in Kobe. The hospital also has eight beds in its medical psychiatric unit (MPU). During the pandemic period, a state of emergency or semi‐emergency was issued four times in Hyogo Prefecture. The study included 201 participants, however, due to the shortage of beds during the pandemic, we cannot rule out the possibility that emergency inpatients were discharged by their attending physicians without a psychiatrist's consultation. Two hundred one patients visited the emergency room and were hospitalized for suicide attempts, met our inclusion criteria and were further analyzed; Table 1 shows the patient characteristics. The number of patients admitted to Kobe City Medical Center General Hospital with COVID‐19 was 1368. In both periods, more women visited the emergency room after suicide attempts compared to men. There was no significant difference in patients' mean age before and during the COVID‐19 pandemic. Physical complication rates before and during the pandemic were significantly different (*p* < 0.001); however, the CCI score was not (*p* = 0.054). Acute drug intoxication during the COVID‐19 pandemic was significantly more common as compared to before (*p* = 0.006). Similarly, overmedication as a means of self‐injury was significantly higher during the pandemic compared to before (*p* = 0.023). Self‐inflicted injuries with high fatality rates (suicide by hanging, jumping, diving, cervical stab wounds, abdominal stab wounds, falls, and toxic gas) decreased during the pandemic; however, the difference was not significant (*p* = 0.227). No significant differences were found between the pre‐ and post‐COVID‐19 periods for either psychiatric illnesses on admission at the hospital or previous psychiatric diagnoses. Employment significantly decreased during the pandemic (*p* = 0.042).

**TABLE 1:** Comparison of patients' characteristics before and during the COVID‐19 pandemic.

	Pre‐COVID‐19 period (*n* = 100)		COVID‐19 period (*n* = 101)		*p*
Age, mean (SD) (years)^a^	43.0 [19.4]		45.3 [22.6]		0.433
Age range (years)	13–79		14–90		
Men, *n* (%)^b^	39	39.0%	43	42.6%	0.667
Occupation					
Unemployed, *n* (%)^c^	69	69.0%	55	54.5%	0.042*
Employed, *n* (%)^c^	31	31.0%	46	45.5%	0.042*
Student, *n* (%)^c^	11	11.0%	17	16.8%	0.309
Skilled/technical, *n* (%)^c^	6	6.0%	11	10.9%	0.311
Office worker, *n* (%)^c^	12	12.0%	9	8.9%	0.499
Heath care worker, *n* (%)^c^	2	2.0%	5	5.0%	0.445
Others, *n* (%)^c^	0	0.0%	4	4.0%	0.121
Family Cohabitants, *n* (%)^c^	74	74.0%	63	62.4%	0.096
Psychiatric history					
Depression, *n* (%)^c^	19	19.0%	18	17.8%	0.857
Bipolar disorder, *n* (%)^c^	13	13.0%	11	10.9%	0.499
Schizophrenia, *n* (%)^c^	12	12.0%	10	9.9%	0.659
Anxiety disorder, *n* (%)^c^	6	6.0%	4	4.0%	0.537
Insomnia, *n* (%)^c^	2	2.0%	5	5.0%	0.445
Personality disorder, *n* (%)^c^	2	2.0%	3	3.0%	1.00
Dissociative disorder, *n* (%)^c^	0	0.0%	3	3.0%	0.246
Intellectual disability, *n* (%)^c^	1	1.0%	3	3.0%	0.621
Others, *n* (%)^c^	21	21.0%	12	11.9%	0.090
None, *n* (%)^c^	24	24.0%	30	29.7%	0.427
Psychotropic medication history					
Antipsychotic drug use, *n* (%)^c^	45	45.0%	46	45.5%	1.00
Antidepressant use, *n* (%)^c^	28	28.0%	21	20.8%	0.254
Anxiolytic and sleep medication (benzodiazepines) use, *n* (%)^c^	49	49.0%	61	60.4%	0.120
History of suicide attempts, *n* (%)^c^	57	57.0%	47	46.5%	0.159
Physical comorbidities, *n* (%)^c^	14	14.0%	38	37.6%	<0.001*
Charlson Comorbidity Index, points (*SD*)^a^	0.17 [0.47]		0.40 [1.06]		0.054
COVID‐19 infection, *n* (%)^c^	‐	‐	6	5.9%	0.029*
Psychiatric diagnosis at admission					
Adjustment disorder, *n* (%)^c^	18	18.0%	19	18.8%	1.00
Schizophrenia, *n* (%)^c^	16	16.0%	13	12.9%	0.553
Depression, *n* (%)^c^	13	13.0%	14	13.9%	1.00
Bipolar disorder, *n* (%)^c^	12	12.0%	11	10.9%	0.828
Personality disorder, *n* (%)^c^	12	12.0%	8	7.9%	0.357
Alcoholism, *n* (%)^c^	8	8.0%	10	9.9%	0.806
Mental retardation, *n* (%)^c^	5	5.0%	8	7.9%	0.568
Dissociative disorder, *n* (%)^c^	3	3.0%	6	5.9%	0.498
Others, *n* (%)^c^	13	13.0%	12	11.9%	0.834
With psychotic symptoms	31	31.0%	38	37.6%	0.373
Diagnosis on admission					
Acute drug intoxication, *n* (%)^c^	51	51.0%	71	70.3%	0.006*
(Multiple) Bone fracture, *n* (%)^c^	20	20.0%	15	14.9%	0.358
Cut wound (chest, abdomen, neck), *n* (%)^c^	11	11.0%	12	11.9%	1.00
Hypoglycemia, *n* (%)^c^	3	3.0%	0	0.0%	0.121
Carbon monoxide poisoning, *n* (%)^c^	3	3.0%	0	0.0%	0.121
None, *n* (%)^c^	7	7.0%	2	2.0%	0.101
Others, *n* (%)^c^	5	5.0%	0	0.0%	0.029*
Admission to medical psychiatric unit, *n* (%)^c^	28	28.0%	23	22.8%	0.421
Intervention by psychiatric liaison team, *n* (%)^c^	56	56.0%	47	46.5%	0.205
Outcome					
Sent home, *n* (%)^c^	63	63.0%	66	65.3%	0.770
Transferred to a psychiatric hospital, *n* (%)^c^	28	28.0%	19	18.8%	0.136
Transferred to a rehabilitation hospital, *n* (%)^c^	9	9.0%	16	15.8%	0.199

*Note*: Pre‐COVID‐19 period: 100 patients comprising inpatients for suicide attempts were provided interventions by psychiatrists from April 1, 2018 to March 31, 2020. All information is noted as investigated in the medical records database. The Charlson Comorbidity Index was calculated using records from the Japanese Diagnosis Procedure Combination data. COVID‐19 period: 101 patients comprised inpatients for suicide attempts, who were provided interventions by psychiatrists from April 1, 2020 to March 31, 2022. *SD*, standard deviation.

* Statistically significant difference (*p* < 0.05).

^a^ Analyzed by the *t*‐test.

^b^ Analyzed by the Chi‐square test.

^c^ Analyzed using Fisher's exact probability test.

Six (5.9%) patients were COVID‐19 positive (*p* = 0.029). Five of these patients (83.3%) had psychosis: one had schizophrenia, two had bipolar disorder, and two had depression. There was a significant difference in psychosis between the patients with and without COVID‐19 (33 of 95 patients; *p* = 0.022). One patient with bipolar disorder had a concomitant developmental disorder. Two patients with depression were the first episode. A significant difference was observed for multiple fractures by diagnosis on admission, with three patients (50.0%) positive for COVID‐19 and 12 patients (12.6%) negative (*p* = 0.041). Five of six patients were students or employed, two of whom were hesitant to talk to others about their COVID‐19 infection for fear of causing trouble to them. Consequently, the infection spread, and they attempted suicide out of remorse. Five of six patients had no history of suicide attempts; however, four had a history of psychiatric treatment, with three continuing it. Two (33.3%) of six patients were admitted to the MPU, and four (66.7%) had a liaison team intervention. Five (83.3%) of six patients had no history of suicide attempts, and none had any complications. Five (83.3%) of six patients were students or employed; 54 (56.8%) COVID‐19 negative patients were unemployed; however, the difference was not significant (*p* = 0.090). Four (66.7%) participants lived with their family members. Comparisons among COVID‐19 negative patients are shown in Table 2.

**TABLE 2:** Comparison of inpatients' characteristics who attempted suicide according to their COVID‐19 status on admission.

	COVID‐19 positive (*n* = 6)		COVID‐19 negative (*n* = 95)		*p‐*value
Age, mean (SD) (years)^a^	32.0 (12.9)		46.2 (23.0)		0.043*
Age range (years)	19–55		14–90		
Men, *n* (%)^b^	1	16.7%	42	45.7%	0.236
Occupation					
Unemployed, *n* (%)^c^	1	16.7%	54	56.8%	0.090
Employed, *n* (%)^c^	5	83.3%	41	43.2%	0.090
Student, *n* (%)^c^	1	16.7%	16	16.8%	1.00
Skilled/technical, *n* (%)^c^	1	16.7%	10	10.5%	0.509
Office worker, *n* (%)^c^	2	33.3%	7	7.4%	0.088
Healthcare worker, *n* (%)^c^	1	16.7%	4	4.2%	0.269
Others, *n* (%)^c^	0	0.0%	4	4.2%	1.00
Family Cohabitants, *n* (%)^c^	4	66.7%	59	62.1%	1.00
Psychiatric history					
Depression, *n* (%)^c^	1	16.7%	17	17.9%	1.00
Bipolar disorder, *n* (%)^c^	0	0.0%	11	11.6%	1.00
Schizophrenia, *n* (%)^c^	1	16.7%	9	9.5%	0.474
Anxiety disorder, *n* (%)^c^	1	16.7%	3	3.2%	0.220
Insomnia, *n* (%)^c^	0	33.3%	5	5.3%	1.00
Personality disorder, *n* (%)^c^	1	16.7%	3	3.2%	0.220
Dissociative disorder, *n* (%)^c^	0	0.0%	3	3.2%	1.00
Mental retardation, *n* (%)^c^	0	0.0%	3	3.2%	1.00
Others, *n* (%)^c^	0	0.0%	13	13.7%	1.00
None, *n* (%)^c^	2	33.3%	28	29.5%	1.00
Psychotropic medication history					
Antipsychotic drug use, *n* (%)^c^	2	33.3%	44	46.3%	0.686
Antidepressant use, *n* (%)^c^	2	33.3%	19	20.0%	0.602
Anxiolytic and sleep medication (benzodiazepines) use, *n* (%)^c^	2	33.3%	59	62.1%	0.210
History of suicide attempts, *n* (%)^c^	1	16.7%	46	48.4%	0.211
Physical comorbidities, *n* (%)^c^	0	0.0%	38	0.4%	0.081
Charlson Comorbidity Index, points (*SD*)^a^	0.0 [0.0]		0.42 [1.09]		<0.001*
Psychiatric diagnosis at admission					
Adjustment disorder, *n* (%)^c^	0	0.0%	19	20.0%	0.591
Schizophrenia, *n* (%)^c^	1	16.7%	12	12.6%	0.572
Depression, *n* (%)^c^	2	33.3%	12	12.6%	0.193
Bipolar disorder, *n* (%)^c^	2	33.3%	9	9.5%	0.127
Personality disorder, *n* (%)^c^	1	16.7%	7	7.4%	0.399
Alcoholism, *n* (%)^c^	0	0.0%	10	10.5%	1.00
Intellectual disability, *n* (%)^c^	0	0.0%	8	8.4%	1.00
Dissociative disorder, *n* (%)^c^	0	0.0%	6	6.3%	1.00
Others, *n* (%)^c^	0	0.0%	12	12.6%	1.00
With psychotic symptoms	5	83.3%	33	34.7%	0.022*
Diagnosis on admission					
Acute drug intoxication, *n* (%)^c^	2	33.3%	63	66.3%	0.183
(Multiple) Bone fracture, *n* (%)^c^	3	50.0%	12	12.6%	0.041*
Cut wound (Chest, abdomen, neck), *n* (%)^c^	0	0.0%	12	12.6%	1.00
None, *n* (%)^c^	0	0.0%	2	2.1%	1.00
Others, *n* (%)^c^	1	16.7%	6	6.3%	0.358
Admission to medical psychiatric unit, *n* (%)^c^	2	33.3%	21	22.1%	0.617
Intervention by psychiatric liaison team, *n* (%)^c^	4	66.7%	43	45.3%	0.413
Outcome					
Home, *n* (%)^c^	3	50.0%	63	66.3%	0.415
Transferred to a psychiatric hospital, *n* (%)^c^	1	16.7%	18	18.9%	1.00
Transferred to a rehabilitation hospital, *n* (%)^c^	2	33.3%	14	14.7%	0.241

*Note*: COVID‐19 positive: Six patients who were inpatients for suicide attempts with COVID‐19 were attended by psychiatrists from April 1, 2020 to March 31, 2022. All information is noted as investigated in the medical records database. The Charlson Comorbidity Index was calculated using records from the Japanese Diagnosis Procedure Combination data. COVID‐19 negative: Ninety‐five patients without COVID‐19 were inpatients for suicide attempts, attended by psychiatrists during the same period. *SD*, standard deviation.

* Statistically significant difference (*p* < 0.05).

^a^ Analyzed by the *t*‐test.

^b^ Analyzed by the Chi‐square test.

^c^ Analyzed using Fisher's exact probability test.

## 4 DISCUSSION

The number of people who visited a hospital after a suicide attempt in the COVID‐19 period (*n* = 101) did not change compared with the pre‐COVID‐19 period (*n* = 100). No significant differences were found in sex or age. The number of suicide attempts owing to overdoses of prescribed medicines, such as sleeping pills, was higher during the COVID‐19 period compared to before. However, the number of all emergency patients, outpatients, and inpatients including suicide visits at the hospital has been decreasing each year at the Kobe City Medical Center General Hospital.^18^ The number of emergency patients peaked at 35 244 in FY2017 and has been declining every year. The lowest was 18 330 in FY2020 during the COVID‐19 pandemic, which increased slightly to 21 230 in FY2021; however, it remains lower than before the COVID‐19 outbreak. These lower rates are likely partly due to hospitals being overwhelmed and reducing services for self‐harm and suicide. Especially at the beginning of the outbreak, on April 9, 2020, nosocomial infections occurred among seven inpatients and 29 staff members, and 349 employees were requested to standby at home in quarantine to prevent the spread of infection,^19^ which had stopped accepting emergency patients except outpatients of our hospital from April 13, 2020. Thus, the number of patients with suicidal behavior visiting emergency rooms is likely to have increased relatively.

Overdoses exhibited a significant increase during the pandemic compared to before, which may be due to stay‐at‐home orders in order to prevent the spread of infection.^20^ These results contradict similar reported statistics showing a decrease in self‐harm during the pandemic period in the United States (March 16, 2020 to October 10, 2020) and Canada (April 1, 2020 to June 30, 2021) when compared to before.^10^, ^11^ This may be because, in Japan, the restrictions were not as strict as the lockdowns in the United States and Canada; for example, there were mild requests for self‐restraint, and people were allowed to go out if they met the requirements. This is the unique mentality of the Japanese. Japanese people are disposed to be attentive and sympathetic toward their surroundings, and traditionally have a sense of group cohesiveness or group consciousness that distinguishes between insiders and outsiders.^21^ This has propelled discrimination against those who are walking outside and suspected of spreading it, which may have influenced the overmedication results.

The high rate of physical complications during the pandemics may be due to the hesitation of accessing hospitals for fear of infection or restrictions on regular hospital care. The number of emergency and outpatient visits has declined since the pandemic slowed, which may indicate that hospital visits in the absence of widespread vaccination may indicate a fear of COVID‐19 infection. In Japan, temporary inoculation for healthcare workers began on February 17, 2021, inoculation for the elderly began on April 12, 2021, and the target age for inoculation was changed from “16 years and older” to “12 years and older” on June 1, 2021. Few people in Japan were able to be vaccinated in FY2020. The use of telemedicine, such as telephone consultation and online medical care, has started. Increased physical anxiety may exacerbate mental health conditions and lead to suicide attempts. Severe physical complications were more common during the COVID‐19 pandemic than before. In contrast, the CCI, a score used to calculate predicted mortality based on comorbidities at admission, did not differ significantly, although it did increase. One possible reason could be that the patients recognized the symptoms but could not consult a doctor before admission, and a severe physical illness may have been discovered after emergency admission for a suicide attempt. Five such patients were identified during the COVID‐19 pandemic.

The increase in the percentage of employed and student patients may be related to anxiety about the future, such as retaining or finding employment, due to the worsening employment situation and economic conditions during the COVID‐19 pandemic and at present. Changes in the number of employed persons revealed a significant decrease among both men and women in April 2020 (when the state of emergency was declared) compared to the previous month. Emergency room visits owing to suicide attempts increased among young adults,^22^ especially girls in Canada.^23^ Economic and employment insecurity raises suicide risk.^24^ There was a higher decrease in the number of female employed persons (with a decrease of 700 000), compared to males (a decrease of 390 000) during the same time period (April 2020).^25^ Therefore, Japanese women may have encountered more financial problems during the pandemic than men. In addition, stay‐at‐home orders may have led to additional stress for women having to spend more time with their husbands and children. Japanese women are characterized by their multiple roles at home, and the rising employment rate of women may lead to increased suicide rates owing to their increased burden.^26^

Although our survey did not find significant differences in mean age or sex, it is reasonable to assume that the relative number of patients was still increasing in Japan, with people typically refraining from consulting a doctor, and the number of patients was decreasing. Although our results do not indicate a definitive increase in suicides during COVID‐19 pandemics, we cannot conclude that the number of people attempting suicide, including youth, young adults, and women, is not increasing.

No significant differences were found in either the intervention rate of the liaison team or the MPU admission rate. Therefore, there appears to be no change in the rate of serious illness. This may have been due to the relative increase in the number of suicides, the extended hospital stay of patients with COVID‐19 infection, and the difficulty in coordinating transfers due to infection control at the transferring hospital, which resulted in fewer available beds. In particular, the MPU has only eight beds, and patients with both severe mental and physical illnesses cannot be transferred immediately, so their length of stay is also longer than in the general wards, which may lead to these results.

Our results indicate that COVID‐19 positive patients who attempted suicide have more severe mental and physical conditions than COVID‐19 negative patients. However, these results should be carefully interpreted as the number of patients was small. COVID‐19 infection may have occurred in the included patients during the COVID‐19 pandemic due to the following: (1) the patients could not divulge their infection status and fluctuating mood disorders led to increased guilt; (2) the patients may have developed depression owing to postinfection sequelae; and (3) the patients originally had severe mental disorders and acted without understanding the risk of infection, which led to infection. COVID‐19 pneumonia with delirium has a high complication rate of encephalopathy^27^ and cognitive dysfunction, fatigue, and neurological symptoms can occur following resolution of acute COVID‐19.^28^ A survey of the effects 6 months after COVID‐19 infection showed that the risk of developing anxiety disorders and other mental disorders was high,^29^ and even after 2 years, where 6% of the patients still had depressive symptoms.^30^

The number of suicides in Japan has been increasing since 1998, peaking at 34 427 in 2003. Therefore, the Japanese government has been making efforts to reduce the suicide rate even before the COVID‐19 pandemic. As a strategic study for suicide countermeasures, a multicenter collaborative randomized comparative study was conducted.^31^ Consequently, suicide prevention and economic stimulus measures were implemented to reduce the number of suicides to 20 169 in 2019 in general. In the current situation, where COVID‐19 is widespread, it can be difficult to provide ongoing mental health support. Unfortunately, the number of suicides may increase due to the pandemics. This is consistent with the report mentioned above.^8^

Conversely, our results showed no changes in the number of suicide attempts before and during COVID‐19. This may be because complying with requests for self‐restraint and behavioral restrictions leads to an increased time spent with the family, which reduced psychological stress and may have contributed to a lower suicide rate.^32^ While there may be a difference between those who attempted suicide and those who have already attempted suicide, the suicide rate was higher than the national average in the areas affected by the Great East Japan Earthquake before the COVID‐19 outbreak but declined during the pandemic.^33^ As an area affected by various disasters, including the 1995 Great Hanshin‐Awaji Earthquake^34^ and the 2009 H1N1 influenza pandemic,^35^ Kobe's suicide rate may have decreased because of disaster‐related mental health approaches to high‐risk groups for suicide. The suicide rate may have remained the same because the general public and healthcare workers have dealt with this pandemic patiently. In fact, a comparison of the 2 years before and during the pandemic shows that the number of suicides in Hyogo Prefecture has decreased from 1831 to 1804, especially among women, from 593 to 584.^36^

If the pandemic is prolonged and affects the public and the economically and socially vulnerable, suicide will become a more pressing issue. It is also possible that domestic violence and alcoholism are becoming increasingly serious, even though they have not yet surfaced.^37^ Suicide prevention measures should be implemented.

### 4.1 Limitations

This study had certain limitations. First, it was based on data from a single institution and an emergency room‐based sample data, which is unlikely to be representative of the entire population. In addition, because this institution accepts all types of patients 24 h a day, 365 days a year, more patients with severe diseases will inevitably be included. Therefore, it might not reflect the overall picture of all emergency patients in Japan. Second, the high severity of suicide attempts among patients with COVID‐19 infection may be a confounding factor, as there may be an association between the risk of COVID‐19 infection and the severity of suicide attempts. A larger, multicenter study is needed to examine the association between COVID‐19 infection and suicide attempts to gain further insight.

## 5 CONCLUSION

It was assumed that the COVID‐19 pandemic and the resulting social unrest would increase people's anxiety and stress, thereby increasing the suicide rate; however, no such signs are currently observed in Kobe's emergency department. It is necessary to predict how people will behave in the future when the COVID‐19 pandemic is declared over in Japan, and behavioral restrictions, both physical and psychological, are no longer in place. In addition, to prevent suicide attempts, it is necessary to establish cooperation between government agencies and relevant community organizations and to develop early response and follow‐up management for those who visit the emergency room after a suicide attempt.

## AUTHOR CONTRIBUTIONS

KO contributed to conceptualization, methodology, and writing–original draft preparation. RY, AN, and KM contributed to methodology. HF contributed to methodology and writing–reviewing and editing.

## FUNDING INFORMATION

This research did not receive any specific grant from funding agencies in the public, commercial, or not‐for‐profit sectors.

## CONFLICT OF INTEREST STATEMENT

The authors declare no conflict of interest.

## ETHICS STATEMENT

Approval of the Research Protocol by an Institutional Reviewer Board: The study protocol was approved by the Research Ethics Review Board of Kobe City Medical Center General Hospital (number: zn220702).

Informed Consent: An opt‐out method was used.

Registry and the Registration No. of the study/trial: N/A.

Animal Studies: N/A.

## Data Availability

The institutional review board of the ethics committee of Kobe City Medical Center General Hospital set restrictions on data sharing because the data contain potentially identifying or sensitive participant information. The database was approved by the research ethics committee. The data that support the findings of this study are available on request from the corresponding author.
